# Is useful research data usually shared? An investigation of genome-wide association study summary statistics

**DOI:** 10.1371/journal.pone.0229578

**Published:** 2020-02-21

**Authors:** Mike Thelwall, Marcus Munafò, Amalia Mas-Bleda, Emma Stuart, Meiko Makita, Verena Weigert, Chris Keene, Nushrat Khan, Katie Drax, Kayvan Kousha

**Affiliations:** 1 Statistical Cybermetrics Research Group, University of Wolverhampton, Wolverhampton, United Kingdom; 2 MRC Integrative Epidemiology Unit at the University of Bristol, Bristol, United Kingdom; 3 School of Psychological Science, University of Bristol, Bristol, United Kingdom; 4 JISC, London, United Kingdom; Universidad de las Palmas de Gran Canaria, SPAIN

## Abstract

Primary data collected during a research study is often shared and may be reused for new studies. To assess the extent of data sharing in favourable circumstances and whether data sharing checks can be automated, this article investigates summary statistics from primary human genome-wide association studies (GWAS). This type of data is highly suitable for sharing because it is a standard research output, is straightforward to use in future studies (e.g., for secondary analysis), and may be already stored in a standard format for internal sharing within multi-site research projects. Manual checks of 1799 articles from 2010 and 2017 matching a simple PubMed query for molecular epidemiology GWAS were used to identify 314 primary human GWAS papers. Of these, only 13% reported the location of a complete set of GWAS summary data, increasing from 3% in 2010 to 23% in 2017. Whilst information about whether data was shared was typically located clearly within a data availability statement, the exact nature of the shared data was usually unspecified. Thus, data sharing is the exception even in suitable research fields with relatively strong data sharing norms. Moreover, the lack of clear data descriptions within data sharing statements greatly complicates the task of automatically characterising shared data sets.

## Introduction

Research data sharing is increasingly encouraged by funders and journals on the basis that data reuse can improve research efficiency and transparency [1,2]. For example, sharing raw data is “strongly encouraged” within open access Plan S (www.coalition-s.org). Shared data may be used to check published findings, for new studies [3], for educational purposes [4] or to support further analyses [5]. Policy initiatives, field cultures and data infrastructure all help to encourage data sharing [6] and researchers seem increasingly willing to publish their data [7]. This may generate citations to the data, originating paper or authors to recognise this effort [8–13], which is a useful incentive [14].

The main disincentives for data sharing are a lack of access to technology or skills [15], the effort needed for curation, fears about prior publication by other researchers [16–19] and low potential for reuse in some fields [4], especially for complex non-standard datasets [20]. The nature and extent of data sharing is highly field dependant [21]. Some fields facilitate sharing with specialised data repositories and/or standards for recording complex data (e.g., [22]). For evolutionary biology, the Dryad repository and journal data sharing mandates have combined to make data sharing almost universal in the top journals [23] (see also [24]). Multidisciplinary generic data sharing policies from publishers can also work reasonably well, with a study of PLoS ONE finding that most articles shared data, albeit with large disciplinary differences in format and sharing method [25]. Nevertheless, most shared ecology and evolutionary research datasets are unable to be reused due to incompleteness or practical difficulties [26]. Biodiversity datasets can be large and hybrid, combining multiple sources. The creation and citing of such datasets are supported by the Global Biodiversity Information Facility, a well-known biodiversity archive, which allows downloads of subsets of data across multiple previously published datasets and creates single DOIs to point to those subsets [27]. These may originate from unpublished work, such as routine data collection exercises or voluntary sharing [28,29], creating data quality validation concerns [30,31]. Even in fields with sharing cultures for standardised data, relatively unique datasets may not be shared, however [32]. Thus, it would be useful to know whether data sharing is widespread in conditions where it has clear value and open data publishing is supported by a research community. This is the first issue addressed in the current paper.

Data sharing may be inadequate for data reuse. The FAIR (findability, accessibility, interoperability, reusability) principles for data sharing emphasise that minimal sharing may not be effective [33]. In particular, techniques for sharing data are not widely standardised and so it is not clear whether it is possible to automatically check the extent to which data sharing occurs in any field and whether the data sharing is effective in the sense of clearly providing sufficient information for others to access and understand it. There is disciplinary diversity in the extent to which communities organise to share data effectively, sometimes driven by external imperatives. For example, some areas of social science have long established effective repositories and standard metadata to facilitate discovery and reuse, archaeologists have more recently started to share data systematically, partly in response to legal restrictions on moving artefacts from their country of origin, but are not well organised. Interviews with researchers about data shared from repositories show that the quality of the documentation is important, as is the reputation of the repository [34]. The ability to automatically extract information about shared data to help its reuse, for example in repositories, is the second issue addressed in the current paper.

Data sharing has been increasing in biomedical science for a long time [35,36], with genomics being regarded as “a leader in the development of infrastructure, resources and policies that promote data sharing” [37] (see also [38]). Pre-publication data sharing has also been advocated in this area [39], and there are even data access committees that judge whether a team should be given access to genomic data from a controlled repository [40,41] (the “gatekeeper” model of data sharing). Within genomics, human Genome-Wide Association Studies (GWAS) seem to be particularly suitable for data sharing. These studies measure the association of genetic anomalies, generally in the form of single nucleotide polymorphisms (SNPs), across the human genome with a potentially inherited characteristic or trait of interest, such as obesity. For each individual location tested on the genome the core result is an effect size coefficient (e.g., odds ratio), standard error and corresponding *p* value derived from a test for whether a particular allele occurs more frequently among (typically) individuals in a risk group compared with a control group. The power of a test is dependent on a sample size so if two or more studies share their data and it is subsequently combined then additional SNPs may be identified [42]. Other analyses are also possible with shared summary GWAS data alone, such as cross-trait linkage disequilibrium score regression [43]. In addition, combining analyses of samples with different control groups enables more universal patterns to be discovered. This is important because many traits are influenced by multiple genes and so different sets of characteristics may produce similar outcomes in different populations. GWAS meta-analysis has evolved as a standard strategy to deal with these issues [44], although it does not seem to be widely used with shared data yet. This is slightly different from the more generic data sharing benefit of sample size increasing statistical power [45].

The GWAS data collection process is expensive and it can be time consuming due to the involvement of human subjects from which tissue samples must be taken. Thus, any data reuse has the potential to provide substantial savings in cost and time. GWAS data sharing has been mandated since January 2008 in NIH-funded research in a specific policy for this study type [46]. In practical terms, GWAS data sharing might be relatively straightforward because the key data is simple (tables of coefficients, standard errors and p values, listed against positions in the human genome using standard notation) and in large consortia, data will need to be internally shared for combining, ensuring that it is typically already in a standard format. The importance of sharing GWAS summary statistics is underlined by the existence of two supportive international databases. Whilst dbGaP (www.ncbi.nlm.nih.gov/gap) allows researchers to deposit this and related data, together with relevant metadata, the GWAS Catalog (www.ebi.ac.uk/gwas) is a manually curated record of the results of GWAS studies [47]. It has hosted GWAS summary statistics since 2017 (www.ebi.ac.uk/gwas/downloads/summary-statistic). GWAS summary statistics never contain personally identifiable information because they are cohort-wide rather than for individuals, and so they may be potentially shared publicly without direct privacy issues, if appropriate human subject permissions have been gained. Nevertheless, it is sometimes possible to estimate the likelihood of a person being present in a cohort from the GWAS summary statistics [48], which is a privacy issue. For detection, the “person’s genotypes for those SNPs and [] a sufficiently representative reference set of allele frequencies” would also be needed [49]. Nevertheless, since many GWAS cohorts have specific diseases, this raises the possibility that a third party with appropriate genomic data could estimate whether a person had a disease when they had contributed to a GWAS study. This led to the NIH recommending controlled access to GWAS data, when shared, but this weakened in 2018 to apply only to sensitive genomic data [50]. Nevertheless, the controversy about the issue may have led researchers to be cautious about any form of data sharing.

We assessed the prevalence of the sharing of GWAS summary statistics in published research and the potential to automatically identify this data using manual checks of 314 primary human GWAS papers from 2010 or 2017, filtered from an initial sample of 1799 papers matching a relevant PubMed query. This topic was chosen as a previously unexplored likely candidate for standardised data sharing, as described above, as well as for being a vibrant research area. For example, the GWAS Catalog (www.ebi.ac.uk/gwas) included statistically significant evidence for 138312 associations with parts of the human Genome by May 2019. The years 2010 and 2017 were chosen to help reveal changes over time. The following research questions encapsulate the broad goals of the project.

1. What proportion of primary human GWAS articles include shared primary GWAS data?
2. Can shared primary GWAS data be automatically identified?

## Methods

A PubMed query was used to identify articles likely to be primary GWAS. PubMed was used since its scope should encompass most GWAS journal articles. A simple MeSH (i.e., Medical Subject Headings controlled vocabulary) query was used rather than a more complex version to enable easier interpretation of the results of the article identification stage. The query was as follows, where the term molecular epidemiology was added to filter out methods-based articles.

### "Molecular Epidemiology"[Majr] AND "Genome-Wide Association Study"[Majr]

After discarding papers that had types other than research-article, this gave 867 journal articles from 2010 and 932 from 2017. The year 2010 was chosen as the first year because there were substantially fewer articles in 2009 and before, whereas there were only small increases after 2010 so 2010 represented the first mature year for GWAS studies. The year 2017 was selected instead of 2018 (in January 2019, at the time of data collection) because there were fewer articles in 2018 than in 2017, suggesting that some PubMed records from this year were missing. The articles were checked for being primary human GWAS by three experienced content analysts by reading their titles, abstract or full text until the classification was clear. The process was as follows.

Articles for non-human genomes were discarded. Articles with the term “meta-analysis” were initially all classed as primary GWAS and then checked by a GWAS expert (MM). In contrast to general meta-analyses, GWAS meta-analyses are usually primary studies that analyse, at least in part, freshly collected data from multiple cohorts. Here, “meta-analysis” refers to the combination of data from multiple sources (i.e., different study samples) rather than a secondary analysis combining data from previously published sources. Articles that were difficult to categorise were forwarded for checking by a GWAS expert (MM). It was not straightforward to check whether an article reported a primary GWAS because it may include both primary and secondary GWAS, it may include prior, parallel or follow-up experiments or analyses, and the details may be described in technical language that avoids the term GWAS within the methods and results. The first author re-checked all articles classified as primary human GWAS. A fifth coder, a GWAS researcher (MM) checked 77 random articles and made 12 corrections, in all cases ruling out an article initially judged to be a primary human GWAS. For example, “Locus category based analysis of a large genome-wide association study of rheumatoid arthritis” had been categorised as a primary human GWAS because the initial coder and follow-up check had not detected that it did not report collecting primary data. The PubMed IDs of the complete set of 1799 articles were then compared to a list of papers registered in the GWAS Catalog (www.ebi.ac.uk/gwas) and published in 2010 and 2017, and all discrepancies were checked by a GWAS researcher (MM).

After identifying an article as primary human GWAS, the same set of three coders attempted to identify whether it shared GWAS summary statistics. This information was first sought in Data Availability statements, if any, or at the end of the article, or in associated supplementary information files. Failing these, the remainder of the article was scanned for references to summary statistics. An article was recorded as sharing summary statistics only if it included a complete set rather than just the statistically significant ones because a full set is needed for reuse. This is because p-values below a statistical significance threshold for one study may become above the threshold when combined with others, or for a different type of analysis [42,44]. In most cases any shared data was not publicly available, and it was not possible to check what was shared, so the sharing scope was inferred from the data sharing statement (e.g., “all data”). In some cases, the GWAS summary statistics were not shared but instead the matched phenotype and genotype data was made available (e.g., on dbGaP). Since this matched data could be used to reconstruct the GWAS summary statistics, such cases were classed as GWAS sharing to be inclusive. If some data was shared online and other data was available from an author then this was classed as author sharing. A fourth coder (the first author) checked these results and extracted the text in each article referring to the summary (or matched phenotype and genotype) data.

As an additional follow-up check for data sharing, articles in the European Bioinformatics Institute GWAS Catalog (www.ebi.ac.uk/gwas) from 2010 and 2017 with public summary statistics were cross-referenced with the main data set investigated and reasons for any differences identified. This revealed some mismatches and one clear mistake. The original search had missed some primary GWAS without MeSH terms and that had not been matched to *Molecular epidemiology* by PubMed. One matching article had been classed as non-primary GWAS in the main dataset thorough human error.

All identified GWAS Summary Statistics were examined for associated metadata. Without effective descriptions, data is harder to reuse [51].

The methods are limited by the initial MeSH query used, which did not match all GWAS studies, and the use of non-expert coders and cross-checker to classify most of the articles. The methods are also limited by not checking the exact nature of shared data when it had to be requested from the authors or a repository. In some cases, reasonable requests might not be granted or the data shared may not include complete GWAS summary statistics. Data sharing outside of article texts, such as on project or author home pages, was also not checked.

## Results

The individual article classifications are available online (http://doi.org/10.6084/m9.figshare.8006585).

### Availability of GWAS summary statistics

Out of all 314 articles classified as primary human GWAS, 13% reported sharing GWAS summary statistics in some form (or “all data” or matched genotype and phenotype records), increasing substantially from 3% in 2010 to 23% in 2017 (Table 1). If an article did not state that its data was shared, it may still be possible to email the authors to access it or the authors may have subsequently deposited it elsewhere after publication. Conversely, data sharing promised by the authors may not materialise in practice (and perhaps rarely does: [52]) and is time limited. In addition, data sharing statements often did not specify the type of data, so those that were offered by email or by request may not include complete GWAS summary statistics.

**Table 1:** Availability of summary statistics in published primary GWAS articles from 2010 and 2017, according to the article text.

GWAS summary statistics availability	2010	%	2017	%	Total	%
On request to the authors	0	0%	15	9%	15	5%
On request via dbGaP	3	2%	5	3%	8	3%
On request via EGA	1	1%	2	1%	3	1%
On request via another portal	0	0%	3	2%	3	1%
Free online without login, plain text	0	0%	12	7%	12	4%
**Total sharing GWAS data**	**4**	**3%**	**37**	**23%**	**41**	**13%**
Broken link or not findable	3	2%	3	2%	6	2%
Not stated in article	145	95%	122	75%	267	85%
**Human GWAS**	**152**	**100%**	**162**	**100%**	**314**	**100%**
Articles checked	867		932		1799	

When data sharing was flagged in an article, a variety of strategies could be used to access it. Most data (29 out of 41 shared) required a permission seeking stage, either directly from the authors or through the database of Genotypes and Phenotypes (dbGaP) or the European Genome-phenome Archive (EGA) or another access-controlled portal, all of which have approval processes that must be completed before the data can be accessed. The summary statistics were open access in 12 cases, with 2 of these 12 cases lacking descriptive metadata. Thus, when shared, some form of data access control is usually employed.

### Descriptions of the availability of GWAS summary statistics

The S1 List contains a complete list of data sharing statements, together with an indication of where they occurred in each article. These were analysed from the perspective of the potential to automatically extract information about whether the GWAS summary statistics were shared (RQ2).

Articles sharing GWAS summary statistics usually reported this in a Data Availability section or similar within the article (36 out of 47, including those with missing data or broken links). In these sections the location of information about shared data should be straightforward to identify because the sections are short and focused on this goal. Other sections used were: Materials and Methods; Methods; Procedures; Results; Footnotes; Supplementary Information. Such information would be more difficult to automatically extract when mentioned in these sections because it would first need to be identified and then delimited from the rest of the hosting section.

Only nine data sharing statements directly described the shared data as GWAS Summary Statistics (bold and italic [blue] in the S1 List), and these used five different phrases (“GWAS summary statistics” x3, “full GWAS summary statistics” x3, “Summary GWAS estimates”, “Summary statistics for the genome-wide association study”, “genome-wide set of summary association statistics”). The following phrases may have referred to genotype data alone: “GWAS statistics” and “Case Oncoarray GWAS data”. The more general term “genotype data” (found in 8 articles) was more common. This term is ambiguous because there are other forms of genetic analysis, and the phrase is likely to refer to raw genotype data rather than summary statistics derived from combining it with phenotype information. Just under half of the articles describe the sharing policy in the most indirect manner possible, with anaphors “datasets” or “data” (used in 19 articles). Since most articles typically employed multiple analyses and might share incomplete datasets (e.g., just the top SNPs identified, or with the results from some study samples removed), a dataset would need to be identified, downloaded and inspected to check whether it contained complete GWAS summary statistics. In some cases, the data sharing link was to a project website containing similar data from multiple studies so article title matching in the target site was needed to identify the correct dataset. Thus, it would be difficult to automatically identify from data sharing statements whether GWAS summary statistics were shared.

## Discussion

The comparative rarity of GWAS summary statistics data sharing confirms that data sharing is not ubiquitous [6]. This data type seems to be suited for data sharing, given broad community support, a repository infrastructure [47] and methodological benefits from sharing [37,42,44], albeit with ethical issues in some cases. The new insight from the current study is that data sharing can be rare in the absence of mandates even when many other factors favour it. Since journal mandates have been successful in other fields that are arguably less fertile because the data is less standard and there is not an obvious use for some types of data [23], this gives strong evidence that data sharing in science generally is unlikely to become universal without strong mandates.

As alluded to above, the results contrast with previous studies that found a majority of PLOS ONE articles (PLOS has a data sharing mandate) to share data [25] and for data sharing to be close to universal for a set of life sciences journals with a data sharing mandate [23]. The large differences are partly because not all journals include a data sharing mandate, and partly because there is some freedom to interpret these mandates. For example, PLOS journals require authors to share “all data underlying” the findings unless “legal or ethical” reasons apply, in which case researchers must describe how the data can be accessed. One PLOS article in the current dataset did not share its GWAS summary statistics because, “restrictions prohibited us from making the minimal data set publicly available,” but reported that “Data will be available upon request to all interested researchers who meet the criteria for access to confidential data via the Institutional Data Access / Ethics Committee,” but it is not clear whether this includes the full GWAS summary statistics. For GWAS summary statistics, “all data underlying” could arguably refer to the statistically significant results since the remainder do not underlie the findings. For example, one paper reported “Data are available” with a repository reference but this did not lead to the full GWAS summary statistics. Also, for a paper with multiple types of data, the authors might interpret “all data” liberally and focus on one aspect of the data, perhaps the one that they believe would be most useful to share. Some of the papers in the current study classified as not sharing GWAS summary statistics had shared some data, despite lacking this key aspect.

Sufficient provenance and descriptive information must be shared for the data to be reused effectively [34] and it is impossible to mandate for this in general. The data descriptions in the current study were difficult for non-expert humans to interpret the type of data that was shared because the language varied, there were multiple types of data in many articles, and the sharing statements were not constructed to give the key information systematically. One of the advantages of a subject-specific repository can be its ability to develop an understanding of the information needed by a community to reuse resources, translating this into a set of required metadata [34]. The lack of anything approaching this for journals made GWAS summary statistics sharing statements difficult to automatically identify and interpret. This highlights the importance of authors submitting to relevant repositories, so that the information necessary for use can be gathered, even if it is accidentally omitted in journal data sharing statements.

## Conclusions

Only 13% of primary human GWAS studies either share or offer to share their summary statistics data in any form. This is low given that genomics is in many ways the leader in data sharing. Moreover, this type of data is standardised, singled out for a NIH sharing mandate (we did not check whether the articles assessed in this study complied with funder mandates), has had recognised sharing value for over a decade, has public archives to host it, and often needs to be shared internally within research consortia. Other than potential human subjects ethics permissions issues, this type of data seems to be a best case for (partly) non-mandatory scientific data sharing, at least with access control due to potential privacy issues. The percentage shared may increase in the future due to the NIH relaxing access control advice for non-sensitive cohorts. Nevertheless, the low percentage suggests that data sharing is unlikely to become near-universal when it is optional. This emphasises the need for policy initiatives to promote data sharing, to extend the current apparently small minority of data sharing practices.

For GWAS as an illustration, formalised data sharing mandates implemented at the journal level would not be effective because GWAS studies can be published in general, health, psychology and medical journals in addition to specialist genetics and genomics journals. Alternative discipline-specific strategies may need to be devised, perhaps including agreements between funders for this type of data.

In terms of automatically identifying specific types of data reported to be shared in articles, the results suggest that in fields where data sharing statements are widely used, it should be possible to extract information about whether any data was shared. Nevertheless, such sections seem to occur in less than 10% of articles in all broad fields of science (see data shared with: [53]) and so this strategy would not be widely effective. It is much more problematic to identify the type of data shared and seems impractical to automate this step. This is because data sharing statements are typically vague about what is shared and there is no single standard or policy adopted by all journals in a specific field regarding what should be included in a data access statement. Descriptions of the exact nature of the data available would not only help automation but also researchers scanning multiple articles to find relevant data for a new study. Thus, if more journals required data sharing statements and employed guidelines to ensure that the shared data was described in detail, or provided virtual rewards [54] for these activities, then this would support the level of automated data discovery that would be necessary to monitor data sharing and systematically identify shared data for later reuse.

## Supporting information

S1 List Data sharing statements extracted from the study. (DOCX)
