# Supplementary material for: Is useful research data usually shared? An investigation of genome-wide association study summary statistics
Source: PLoS One. 2020 Feb 21;15(2):e0229578. doi: 10.1371/journal.pone.0229578 (PMC7034915; doi:10.1371/journal.pone.0229578)
Supplement: S1 List — (DOCX) [file pone.0229578.s001.docx]

# S1 Appendix

# S1 List: Data sharing statements extracted from the study

1. **Authors:** “Data Availability: [] **Data** requests can be made by contacting []”
2. **Authors**: “Data availability: Genotype data of GERA participants are available from the dbGaP (database of Genotypes and Phenotypes) under accession phs000674.v2.p2. This includes individuals who consented to having their data shared with dbGaP. The complete GERA data are available upon application to the KP Research Bank (https://researchbank.kaiserpermanente.org/). The **summary statistics** generated in this study are available from the corresponding authors upon reasonable request. The ***GWAS summary statistics*** for the replication study11 are available from (https://www.dropbox.com/sh/3j2h9qdbzjwvaj1/AABFD1eyNetiF63I5bQooYura?dl¼0)."
3. **Authors:** “Data Availability Statement: A subset of the data that support the findings of this study is publically available via dbGaP (www.ncbi.nlm.nih.gov/gap; accession number phs001265.v1.p1). The **complete dataset** will not be made publicly available due to restraints imposed by the ethics committees of individual studies; requests for data can be made to the corresponding author or the Data Access Coordination Committee (DACCs) of BCAC (http://bcac.ccge.medschl.cam.ac.uk/): BCAC DACC approval is required to access data from studies ABCFS, ABCS, ABCTB, BBCC, BBCS, BCEES, BCFR-NY, BCFR-PA, BCFR-UT, BCINIS, BSUCH, CBCS, CECILE, CGPS, CTS, DIETCOMPLYF, ESTHER, GC-HBOC, GENICA, GEPARSIXTO, GESBC, HABCS, HCSC, HEBCS, HMBCS, HUBCS, KARBAC, KBCP, LMBC, MABCS, MARIE, MBCSG, MCBCS, MISS, MMHS, MTLGEBCS, NC-BCFR, OFBCR, ORIGO, pKARMA, POSH, PREFACE, RBCS, SKKDKFZS, SUCCESSB, SUCCESSC, SZBCS, TNBCC, UCIBCS, UKBGS and UKOPS (see Supplementary Table 1).”
4. **Authors:** “Data availability: The ***full GWAS summary statistics*** for the 23andMe discovery data set may be requested from 23andMe, Inc. and received subject to the execution of 23andMe's standard data transfer agreement, which includes clauses intended to protect the privacy of 23andMe participants, among other matters.”
5. **Authors (also dbGaP and EGA):** “Data availability: The scan IARC-2 obtained Institutional Review Board certification permitting data sharing in accordance with the US NIH Policy for Sharing of Data Obtained in NIH Supported or Conducted GWAS. Data are accessible on dbGaP (study name: ‘Pooled Genome-Wide Analysis of Kidney Cancer Risk (KIDRISK)’; url: http://www.ncbi.nlm.nih.gov/projects/gap/cgi-bin/study.cgi?study_id=phs001271.v1.p1). Similarly, the NCI-1 scan is accessible on dbGaP (phs000351.v1.p1). **Data** from IARC-1 and MDA scans are available from Paul Brennan and Xifeng Wu, respectively, upon reasonable request. The UK **scan data** will be made available on the European Genome-phenome Archive database (accession number: EGAS00001002336). The NCI-2 scan will be posted on dbGaP.”
6. **Authors:** “Data Availability Statement: Please contact author for **data** requests”
7. **Authors:** “Data Availability Statement: The Icelandic population [Whole Genome Sequencing] data have been deposited at the European Variant Archive under accession code PRJEB8636. The authors declare that the **data** supporting the findings of this study are available within the article, its Supplementary Information files and on request.”
8. **Authors:** “Availability of data and materials: The **dataset** generated in AA-DHS are available from the senior author based on reasonable request. JHS [replication] data are available on dbGap and/or direct request addressed to the JHS leadership.”
9. **Authors**: “Data Availability Statement: The top 10K SNPs for five personality traits from the 23andMe discovery data set are available in Supplementary Data Sets 1–5. The ***full GWAS summary statistics*** for the 23andMe discovery data set will be made available through 23andMe to qualified researchers under an agreement with 23andMe that protects the privacy of the 23andMe participants. Please contact [] for more information and to apply for data access.”
10. **Authors**: “Data availability statement: ***GWAS summary statistics*** will be made available for researchers (appendix p 3)." and "Summary statistics for the EU-RLS-GENE GWAS dataset will be made available to qualified researchers. Please contact [] for details and to apply to access the data. Correspondence and requests for materials relating to the UK INTERVAL data set should be addressed to [] and []. Summary statistics for the 23andMe dataset will be made available through 23andMe to qualified researchers under an agreement with 23andMe that protects the privacy of the 23andMe participants. Please contact [] for more information and to apply to access the data. "
11. **Authors**: “Data availability: [] The ***full GWAS summary statistics*** for PDWBS will be made available through 23andMe and Genentech to qualified researchers under an agreement with 23andMe that protects the privacy of the 23andMe participants and an agreement with Genentech for data sharing. Please contact D.H. (dhinds23andme.com) for more information and to apply to access the data.”
12. **Authors**: “Data availability: **All relevant data** are available from the authors and summary level results are available on dbGaP.”
13. **Authors**: “Data Availability: **All data that support the findings of this study** are available from the corresponding author on reasonable request with the exception of results from 23andMe. To request access to the 23andMe summary statistics, please email [].”
14. **Authors**: “Data Availability Statement: The data of the Exomechip cohort is available in dbGap (phs001306.v1.p1). The **GWAS statistics** from the 23andMe cohort can be requested by applying to the 23andMe collaboration program.”
15. **dbGaP**: “Data Availability: The phenotypic data for the Health and Retirement Study (HRS) is public release data to registered users and researchers can become registered users through the University of Michigan (UM) data portal (http://hrsonline.isr.umich.edu/index.php?p=reg&_ga=2.214793619.503473076.1498851443-177726954.1424977713); however, registered users are not permitted to redistribute the data to third parties per the data use agreement (http://hrsonline.isr.umich.edu/index.php?p=regcou). The HRS **genotype data** is available to approved users through the NCBI Database of Genotypes and Phenotypes (dbGaP).”
16. **dbGaP**: [in **Methods** section] “The **datasets** used for the analyses described in this paper can be obtained from the database of Genotypes and Phenotypes (dbGaP) at http://www.ncbi.nlm.nih.gov/projects/gap/cgi-bin/study.cgi?study_id=phs000092.v1.p1 through dbGaP accession number phs000092.v1.p1.”
17. **dbGaP**: “Data Availability Statement: **Genotype data** from the GICC GWAS are available from the database of Genotypes and Phenotypes (dbGaP) under accession phs001319.v1.p1.”
18. **dbGaP:** [in **Methods** section] “**Meta-analysis results** are available on dbGaP (https://www.ncbi.nlm.nih.gov/gap; accession number phs000930)."
19. **dbGaP:** “Data Availability: The primary **data** are available from dbGAP, accession number phs000431.v2.p1.”
20. **dbGaP:** “Data availability: We have deposited all **genotype data** supporting our findings from the discovery cohort in the Database of Genotypes and Phenotypes (dbGaP), with accession code phs000421.v1.p1. Other data that support our findings are available from the authors by request; see author contributions and their published references for specific data sets.”
21. **dbGaP**: “Data accession: The **genotype data** for the 311,459 SNPs in 1215 Behçet's disease cases and 1278 healthy controls from Turkey have been deposited in the National Institutes of Health database of genes and phenotypes, dbGaP (http://www.ncbi.nlm.nih.gov/sites/entrez?db=gap), accession number: phs000272.v1.p1”
22. **dbGaP**: "Data Availability: The **datasets** generated during the current study are available at the URL: https://www.ncbi.nlm.nih.gov/projects/gap/cgi-bin/study.cgi?study_id=phs001273.v1.p1."
23. **dbGaP**: "[in **Footnotes**] NIMH bipolar disorder sample: dbGAP phs000017.v1.p1.c1-c3; GAIN major depression **sample**: dbGAP phs000020.v1.p1"
24. **EGA:** “Data Availability Statement: A list of the SNPs in the discovery scan exhibiting P < 10−4 are available in Supplementary Table 9. Researchers can gain access to the **data** by applying to the data access committee (www.ebi.ac.uk/ega/).”
25. **EGA:** “DATA AVAILABILITY: ***Case Oncoarray GWAS data*** and the Hi-C dataset utilized in this paper have both been deposited in the European Genome–phenome Archive (EGA), which is hosted by the European Bioinformatics Institute (EBI), under the accession codes EGAS00001001836 and EGAS00001001930 respectively.”
26. **EGA:** “Data Availability: All relevant **data** are available from the European Genome Archive (EGA) with the accession number: EGAD00010001447.”
27. **Other portal**: “Data availability: Data, including all **genotype data** and information on hypertension status, are available on approximately 78% of [Genetic Epidemiology Research on Adult Health and Aging] participants from dbGaP under accession code phs000674.v1.p1. This includes individuals who consented to having their data shared with dbGaP. The complete GERA data are available upon application to the KP Research Bank Portal”
28. **Other portal**: “Data availability: Stage one **data** are from UK Biobank, and can be obtained upon application (ukbiobank.ac.uk)”
29. **Other portal:** [in **Materials and Methods** section] "The **raw genotype and phenotype data** of the Tibetan and Han subjects are available through application at https://www.wmubiobank.org".
30. **Open access**: “Data Availability: A **dataset** file is available from the GRASP resources data. The URL is https://grasp.nhlbi.nih.gov/FullResults.aspx. The study will be found using the first author name (Salem) or the pubmed ID.”
31. **Open access:** “Data Availability: All relevant **data** are within the manuscript, supporting information files, and hosted at the following URL: http://cmgm.stanford.edu/~kimlab/ACL/Achilles_ACL.html. Data will also be available at NIH GRASP: https://grasp.nhlbi.nih.gov/FullResults.aspx.”
32. **Open access:** “Data Availability: All relevant **data** can be accessed at NIH GRASP by using the following link: https://grasp.nhlbi.nih.gov/FullResults.aspx.”
33. **Open access**: “Data Availability: All the ***summary level data***, as well as the individual level data for Tanzania are available from DRYAD (doi:10.5061/dryad.cq183)”.
34. **Open access:** “Data Availability: These third party **data** are available from NIH GRASP. The authors did not have any special access privileges and interested researchers can access the data at https://grasp.nhlbi.nih.gov/FullResults.aspx (Trait(s): Ankle injury).”
35. **Open access**: “Data Availability Statement. ***Summary GWAS estimates*** for the T2D meta-analysis and bivariate summary data are publicly available at the following:”
36. **Open access:** “Data availability. The **genotype data**, BMI measurements, and related phenotype information that support the findings of this study are available in Japanese Genotype-phenotype Archive (JGA) under accession codes JGAS00000000114 for the study, JGAD00000000123 for the genotype data, and JGAD00000000124 for the BMI measurements. The summary statistics of the GWAS have been deposited in the National Bioscience Database Center under data set identifier hum0014.v6.158k.v1.”
37. **Open access:** "**Supplementary information**: [] Supplementary Data 2: ***Summary statistics for the genome-wide association study***."
38. **Open access**: “**Footnotes** [] Web resources: ***Summary statistics from the meta-analyses***, <http://computationalmedicine.fi/data#Cytokine_GWAS>”
39. **Open access**: ***GWAS summary statistics*** from this study available via the GEnetic Factors for OSteoporosis Consortium website (http://www.gefos.org/).”
40. **Open access**: “Data Availability Statement: ***Summary statistics*** have been made available for download from <http://ctg.cncr.nl/software/summary_statistics>.”
41. **Open access**: “Data Availability and Accession Code Availability Statements: The **human genotype and phenotype data** on which the results of this study are based are available upon application from the UK Biobank Study (http://www.ukbiobank.ac.uk/).
42. **Missing/broken**: [in **Results** section] "The complete set of ***results from this genomewide association study*** can be found in the National Institutes of Health Genotype and Phenotype database (dbGaP; www.ncbi.nlm.nih.gov/projects/gap/cgi-bin/about.html) (accession number phs000233.v1.p1).”
43. **Missing/broken**: [in **Results** section] “A ***genome-wide set of summary association statistics*** will be available at the National Bioscience Database Center (NBDC)”
44. **Missing/broken**: [in **Results** section] "The **summary of statistical analysis in the first stage** is available on the genome-wide association database (https://gwas.lifesciencedb.jp/cgi-bin/gwasdb/gwas_study.cgi?id=cerebral)."
45. **Missing/broken**: "[**Procedures**] **All data** were deposited at the ALS online genetics database18 [broken] and the European genome-phenome archive. <https://www.ebi.ac.uk/ega/>" [incomplete GWAS]
46. **Missing/broken**: “Data availability statement: ***Summary statistics for all meta-analyses*** will be made available at the following website <https://www.nhlbi.nih.gov/research/intramural/researchers/ckdgen>.”
47. **Missing/broken**: “Data Availability Statement: The ***meta-analysis results from this study*** are available at dbGAP (accession number phs000930).” [incomplete]
